# Toxicity Assessment of SiO_2_ and TiO_2_ in Normal Colon Cells, In Vivo and in Human Colon Organoids

**DOI:** 10.3390/molecules25163594

**Published:** 2020-08-07

**Authors:** Sung Bum Park, Won Hoon Jung, Ki Young Kim, Byumseok Koh

**Affiliations:** Drug Discovery Platform Research Center, Korea Research Institute of Chemical Technology, 141 Gajeong-ro, Yuseong-gu, Daejeon 34114, Korea; spark@krict.re.kr (S.B.P.); whjeung@krict.re.kr (W.H.J.)

**Keywords:** silicon dioxide nanoparticle, titanium dioxide nanoparticle, cytotoxicity, in vivo toxicity, colon organoids

## Abstract

We conducted systemic assessments on the toxicity of silicon dioxide (SiO_2_) and titanium dioxide (TiO_2_) nanoparticles using different forms of normal colon cells (CCD-18Co), in vivo and in human colon organoids. The in vivo acute oral toxicity data showed that the LD_50_ values are greater than 2000 mg/kg for both the SiO_2_ and TiO_2_ nanoparticles; however, the SiO_2_ and TiO_2_ nanoparticles induced cytotoxicity in two-dimensional CCD-18Co cells and three-dimensional CCD-18Co spheroids and human colon organoids, with IC_50_ values of 0.6, 0.8 and 0.3 mM for SiO_2_ and 2.5, 1.1 and 12.5 mM for TiO_2_ nanoparticles, respectively. The data suggest that, when SiO_2_ and TiO_2_ are in nanoparticle form, cytotoxicity is induced; thus, care should be taken with these materials.

## 1. Introduction

Silicon dioxide (SiO_2_) and titanium dioxide (TiO_2_) are food additives [1,2,3]. SiO_2_ usually serves as an anti-clumping agent and prevents powdered ingredients from sticking together [4,5,6]. TiO_2_ is widely used as an anti-whitening agent [7,8,9]. As both are widely used as food additives, the Food and Drug Administration (FDA) provides general guidelines for the human consumption of SiO_2_ and TiO_2_ [10,11]. Both SiO_2_ and TiO_2_ in dietary forms are considered relatively less toxic than other forms and are permitted for human intake when 2% (for SiO_2_) and 1% (for TiO_2_) or less are present in food. However, the toxicity derived from SiO_2_ and TiO_2_ nanoparticles is still unclear and thus needs to be carefully elucidated. Indeed, studies have suggested that both SiO_2_ and TiO_2_ nanoparticles can be present in foods [12,13]. SiO_2_ and TiO_2_ nanoparticles can also induce cytotoxicity and thus may have a negative health impact. These studies have shown that SiO_2_ and TiO_2_ nanoparticles show toxicity in the human lung, intestinal cells and during in vivo experiments [14,15,16,17,18,19].

Human organoids—self-organized three-dimensional (3D) cell cultures that are derived from stem cells or isolated from tissue—can be considered miniaturized and simplified versions of organs [20,21,22,23,24]. Human organoids are often considered one of the most relevant in vitro systems for biological studies because they closely mimic each designated organ. Three-dimensional cell cultures exhibit greater differential potential and are more physiologically relevant and better represent in vivo tissue [25,26]. Therefore, toxicity evaluation using organoids, as well as 3D cell culture, can provide an in-depth understanding of the toxic nature of substances [25,26,27].

Although several studies have shown toxicities induced by SiO_2_ and TiO_2_ in vitro and in vivo [28,29,30], studies on the toxicities of SiO_2_ and TiO_2_ using human colon organoids are rare. In addition, as both SiO_2_ and TiO_2_ are often used as food supplements, systemic studies on the effects of SiO_2_ and TiO_2_ nanoparticles on the human colon should be conducted.

To systemically evaluate SiO_2_ and TiO_2_ nanoparticle-induced cytotoxicity and to fill this research gap, we conducted systemic toxicity studies of SiO_2_ and TiO_2_ nanoparticles in human normal colon fibroblasts, in vivo ICR mouse and in human colon organoids. Different aspects of the toxicities induced by SiO_2_ and TiO_2_ nanoparticles suggest that care should be taken when SiO_2_ and TiO_2_ are in nanoparticle forms.

## 2. Results

### 2.1. Characterization of SiO_2_ and TiO_2_ Nanoparticles

**Figure 1:**
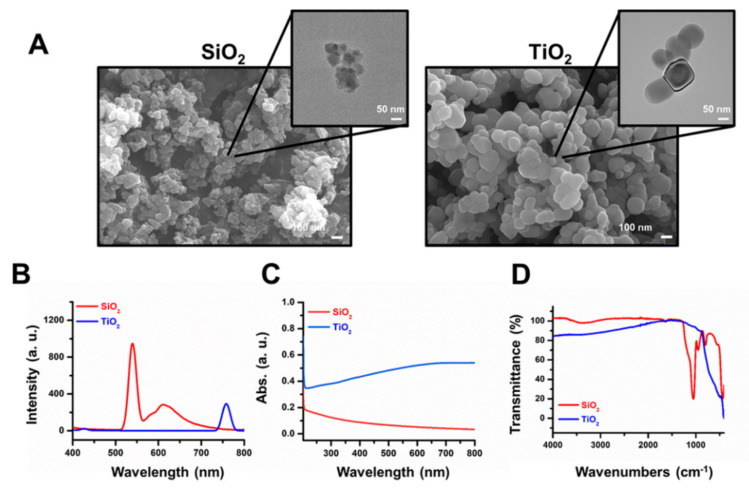
Characterization of the sterilized SiO_2_ and TiO_2_ nanoparticles. (**A**) Scanned electron microscopy (SEM) and transmission electron microscopy (TEM, small picture) images of the SiO_2_ and TiO_2_ nanoparticles. (**B**) PL (1 µM solubilized in ethanol). (**C**) Ultraviolet–visible absorbance spectra (1 mM solubilized in ethanol) and (**D**) Fourier-transform infrared spectra of the SiO_2_ and TiO_2_ nanoparticles.

Scanning electron microscopy (SEM) and transmission electron microscopy (TEM) images showed that both SiO_2_ and TiO_2_ nanoparticles after the sterilization process had a sub-100 nm size on average (Figure 1A). The photoluminescence (PL) spectra showed a peak intensity at 537 nm for the sterilized SiO_2_ nanoparticles and 762 nm for the sterilized TiO_2_ nanoparticles (Figure 1B). The ultraviolet–visible spectroscopy (UV–vis) absorbance spectra showed maximum absorbance at 201 nm and 205 nm for SiO_2_ and TiO_2_ nanoparticles, respectively (Figure 1C), and the Fourier transform infrared spectroscopy (FT-IR) data showed peaks at 1099 cm^−1^ for SiO_2_ and 490 cm^−1^ for TiO_2_ nanoparticles (Figure 1D). The hydrodynamic diameters and polydispersity indexes for SiO_2_ and TiO_2_ nanoparticles were 980 nm, 23.2% and 470 nm, 22.3%, respectively (Table S1). Zeta potentials for SiO_2_ and TiO_2_ nanoparticles were −11.8 and −28.1 mV, respectively (Table S2).

### 2.2. Toxicity in Two-Dimensional (2D) CCD-18Co Cells

**Figure 2:**
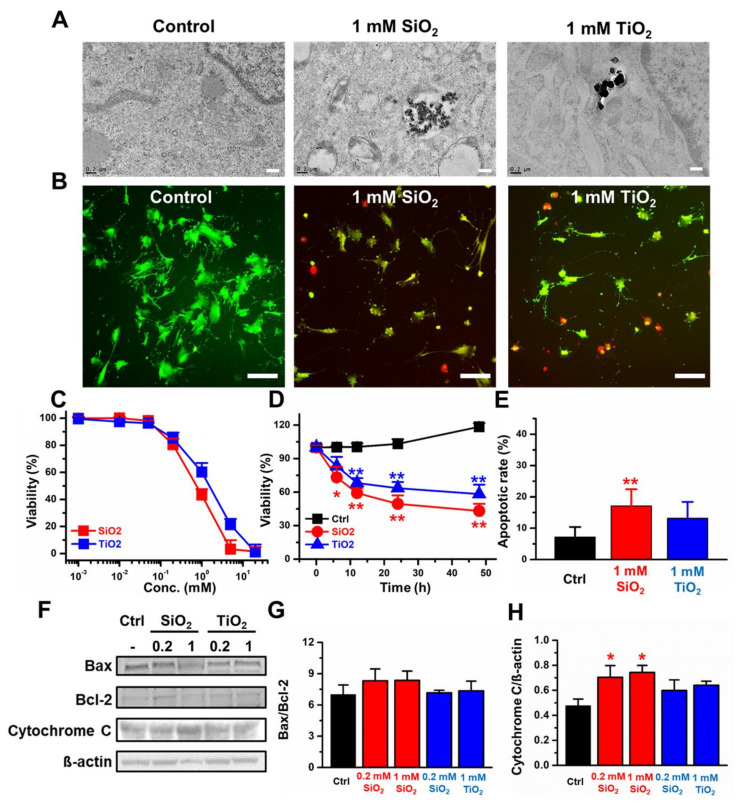
The cytotoxicity induced by SiO_2_ and TiO_2_ nanoparticles in 2D CCD-18Co. (**A**) Transmission electron microscopy (TEM) images of the control and the SiO_2_ and TiO_2_ nanoparticles endocytosed by CCD-18Co cells. The scale bar in represents 0.2 µm. (**B**) Live/dead assay (Green: live cells, Red: dead cells). Scale bars represent 20 µm. (**C**) Concentration-dependent viability. (**D**) Incubation time-dependent viability, (**E**) % cells undergoing apoptosis. (**F**) Western blots. (**G**) Quantitative analysis of the Bax/Bcl-2 protein expression ratio. (**H**) Quantitative analysis of the Cytochrome C/ß-actin ratio of the 2D CCD-18Co cells treated with the SiO_2_ and TiO_2_ nanoparticles. Error bars represent the standard deviation of three replicates. * For *p* < 0.05, ** for *p* < 0.01.

The 2D CCD-18Co cells were incubated with the designated concentrations of SiO_2_ and TiO_2_ to evaluate nanoparticle-induced toxicities. TEM images showed that the SiO_2_ and TiO_2_ nanoparticles were endocytosed in the CCD-18Co cells (Figure 2A). The data suggested that a 24 h incubation with nanoparticles induced CCD-18Co cell death with IC_50_ values of 0.6 and 2.5 mM for the SiO_2_ and TiO_2_ nanoparticles, respectively (Figure 2B,C). The cell viability of CCD-18Co incubated with nanoparticles gradually decreased over 48 h of incubation, with 43.2% and 58.3% remaining viable for 1 mM SiO_2_ and TiO_2,_ respectively (Figure 2D). On average, 17.1% and 13.2% of the 2D CCD-18Co cells underwent apoptosis when treated with 1 mM SiO_2_ and TiO_2_ nanoparticles, respectively, while the control CCD-18Co cells showed only 7.1% apoptosis (Figure 2E). Western blotting of apoptotic markers was performed for the CCD-18Co cells treated with the SiO_2_ and TiO_2_ nanoparticles (Figure 2F). No significant changes in Bax/Bcl-2 ratio was observed when treated with SiO_2_ and TiO_2_ nanoparticles (Figure 2G), however the cytochrome C level increased by around 56.8% and 61.3% when treated with 0.2 and 1 mM of SiO_2_ nanoparticles, respectively (Figure 2H).

### 2.3. Toxicity of the CCD-18Co 3D Spheroids

**Figure 3:**
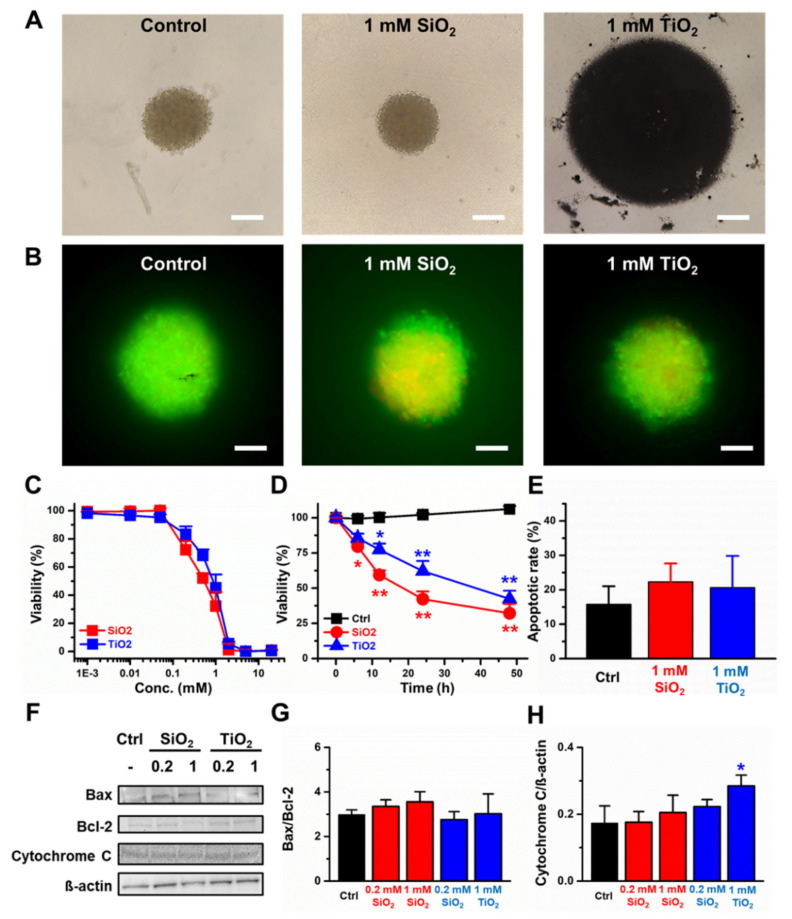
Cytotoxicity induced by the SiO_2_ and TiO_2_ nanoparticles in the 3D CCD-18Co spheroids. (**A**) Bright field images of control-SiO_2_ nanoparticle-and TiO_2_ nanoparticle-incubated CCD-18Co spheroids. The scale bar represents 0.2 µm. (**B**) Live/dead assay (Green: live cells, Red: dead cells). Scale bars represent 20 µm. (**C**) Concentration-dependent viability, (**D**) Incubation time-dependent viability. (**E**) % of cells undergoing apoptosis. (**F**) Western blot. (**G**) Quantitative analysis of the Bax/Bcl-2 protein expression ratio. (**H**) Quantitative analysis of the cytochrome C/ß-actin ratio of 3D CCD-18Co spheroids treated with the SiO_2_ and TiO_2_ nanoparticles. Error bars represent the standard deviation of three replicates. * For *p* < 0.05, ** for *p* < 0.01.

The 3D CCD-18Co spheroids were formed, and the toxicities of the SiO_2_ and TiO_2_ nanoparticles were monitored. The data suggested that both nanoparticles induced cytotoxicity with IC_50_ values of 0.8 and 1.1 mM for the SiO_2_ and TiO_2_ nanoparticles, respectively, after 24 h of incubation (Figure 3A–C). Compared to the control, CCD-18Co 3D spheroids, the spheroids treated with 1 mM SiO_2_ and TiO_2_ nanoparticles showed decreased viability within the designated time frame (48 h; Figure 3D). We examined the rate of cells in the 3D spheroids undergoing apoptosis and found that 15.8% of the control CCD-18Co cells in spheroids underwent apoptosis, while 22.3% and 20.6% of the CCD-18Co spheroids underwent apoptosis when treated with 1 mM SiO_2_ and TiO_2_ nanoparticles, respectively (Figure 3E). Western blot results showed that both the Bax/Bcl-2 and the cytochrome C/ß-actin ratios did not change >20% when treated with 0.2 and 1 mM SiO_2_ and TiO_2_ nanoparticles (Figure 3F–H).

### 2.4. In Vivo Toxicities of the SiO_2_ and TiO_2_ Nanoparticles

**Figure 4:**
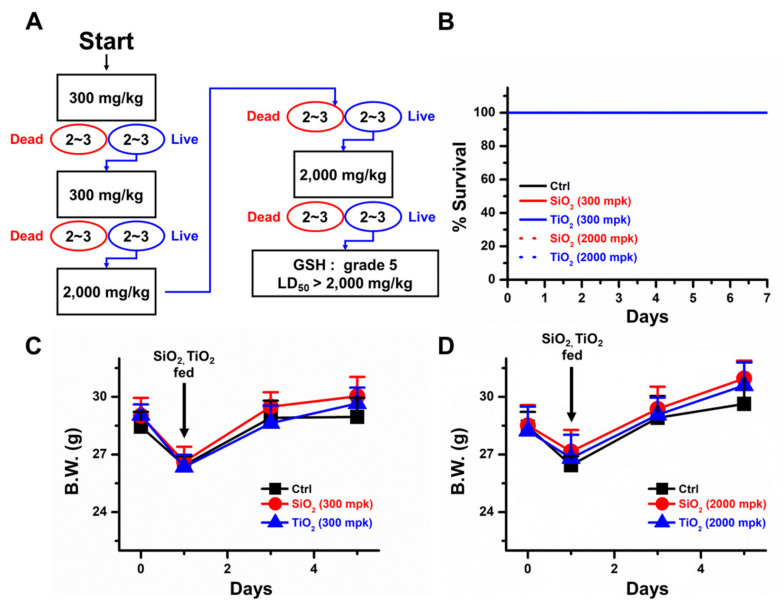
In vivo acute oral toxicity assessment of SiO_2_ and TiO_2_ nanoparticles. (**A**) Toxicity assessment scheme. (**B**) % survival of mice after oral feeding of 300 and 2000 mg/kg SiO_2_ and TiO_2_ nanoparticles. (**C**) Body weight changes induced by 300 mg/kg SiO_2_ and TiO_2_ nanoparticles. (**D**) Body weight changes in the 300 mg/kg SiO_2_ and TiO_2_ nanoparticle-treated mice. Error bars represent the standard deviation of three replicates. Abbreviation mpk is mg/kg.

In vivo acute oral toxicity assessments were conducted to verify the effects of SiO_2_ and TiO_2_ nanoparticle intake in mice. The mice were first orally fed 300 mg/kg SiO_2_ and TiO_2_ nanoparticles in 0.5% carboxymethylcellulose (CMC) solution, and the % survival rate and body weight were monitored (Figure 4A). The data showed that the mice fed 300 mg/kg SiO_2_ and TiO_2_ nanoparticles survived for more than 7 days without notable changes in body weight compared to the control mice (Figure 4B,C). The mice were further fed 2000 mg/kg SiO_2_ and TiO_2_ nanoparticles, and 100% of the mice survived for more than 7 days without significant loss or gain of body weight (Figure 4B,D). Overall, the data suggested that both SiO_2_ and TiO_2_ nanoparticles are globally harmonized classification system (GHS) grade 5, and have lethal dose 50 (LD_50_) values of greater than 2000 mg/kg in mice.

### 2.5. Toxicity Assessment of the SiO_2_ and TiO_2_ Nanoparticles in Human Colon Organoids

**Figure 5:**
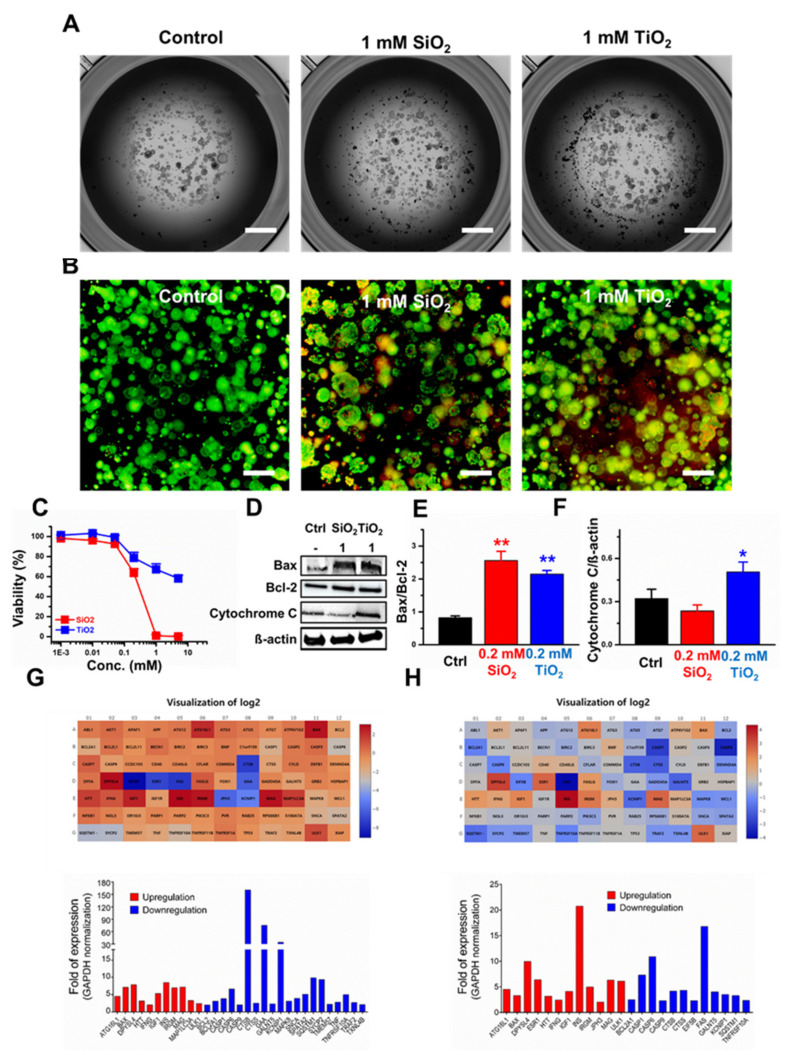
Human colon organoid toxicity induced by the SiO_2_ and TiO_2_ nanoparticles. (**A**) Bright field images of control-SiO_2_ nanoparticle-and TiO_2_ nanoparticle-incubated human colon organoids. The scale bar represents 200 µm. (**B**) Live/dead assay. Scale bars represent 150 µm. (**C**) Concentration-dependent viability. (**D**) Western blot. (**E**) Quantitative analysis of the Bax/Bcl-2 protein expression ratio. (**F**) Quantitative analysis of the cytochrome C/ß-actin ratio of human colon organoids treated with 0.2 mM SiO_2_ and TiO_2_ nanoparticles. Error bars represent the standard deviation of three replicates. (**G**,**H**) Heat maps and fold of mRNA expression level changes after (**G**) 0.2 mM SiO_2_ and (**H**) TiO_2_ treatment. Values are means of pooling data from three separate experiments. Heat map and analysis of expression changes was performed using the software (Qiagen Geneglobe) supplied by Qiagen (https://geneglobe.qiagen.com/kr/analyze). * For *p* < 0.05, ** for *p* < 0.01.

Human colon organoids were treated with the SiO_2_ and TiO_2_ nanoparticles to monitor their toxicity. The data on the SiO_2_ and TiO_2_ nanoparticle concentration-dependent human colon organoid viability showed that increasing the concentrations of SiO_2_ and TiO_2_ nanoparticles induced a decrease in viability with IC_50_ values of 0.3 mM and 12.5 mM for the SiO_2_ and TiO_2_ nanoparticles, respectively (Figure 5A–C). Western blotting was performed to monitor the effects of the SiO_2_ and TiO_2_ nanoparticles on the protein expression of the human colon organoids. The Bax/Bcl-2 ratio increased by 3.1- and 2.6-fold after treatment with the 0.2 mM SiO_2_ and TiO_2_ nanoparticles, respectively (Figure 5D,E), while the cytochrome C/ß-actin ratio decreased by 27% after treatment with the SiO_2_ nanoparticles and increased by 1.6-fold after treatment with the TiO_2_ nanoparticles compared to the control (Figure 5F). Gene expression analysis was performed on human colon organoids treated with 0.2 mM SiO_2_ and TiO_2_ nanoparticles. Thirty-one genes out of 84 were found to have changed expression levels (11 upregulated and 20 downregulated; Figure 5G) in the human colon organoids treated with the SiO_2_ nanoparticles (a fold change > 2.0 and a *p*-value below 0.05). After treatment with the TiO_2_ nanoparticles, 24 genes out of 84 showed changes in expression (12 upregulated and 12 downregulated; Figure 5H). The 84 gene expression changes are shown in Supplementary excel data. As a result, the levels of 10 genes (including Bax and Insulin precursor, Ins) were upregulated in the human colon organoids treated with either the SiO_2_ or TiO_2_ nanoparticles, compared to the control, while the levels of 10 genes (including Bcl2A1 and caspases 1, 6 and 9) were downregulated after treatment with either the SiO_2_ or TiO_2_ nanoparticles compared to the control.

## 3. Discussion

SiO_2_ and TiO_2_ are widely used in food products as carriers for thickeners, anticaking agents, fragrances and flavors. However, problems with the oral uptake of engineered SiO_2_ and TiO_2_ nanoparticles in food have been reviewed recently. Therefore, in this study, we hypothesized that a high amount of SiO_2_ and TiO_2_ nanoparticles could induce toxicity in human colon organoids, which we processed in the experiments.

In this study, SiO_2_ and TiO_2_ nanoparticles were characterized to confirm whether their properties were changed during the sterilization process. The data suggested that autoclaving and UV sterilization do not significantly change the properties of SiO_2_ and TiO_2_ nanoparticles. SEM and TEM images of the SiO_2_ and TiO_2_ nanoparticles showed average diameters of 75.1 and 113.5 nm, respectively. PL, UV-vis absorbance and FT-IR spectra of the sterilized SiO_2_ and TiO_2_ nanoparticles showed that the spectroscopic properties of the nanoparticles did not significantly change from previously reported data after the sterilization processes [31,32,33]. After the characterization of the sterilized SiO_2_ and TiO_2_ nanoparticles, 2D and 3D human normal colon fibroblast CCD-18Co cells were used to monitor their cytotoxicity. TEM images of the 2D CCD-18Co cells incubated with the SiO_2_ and TiO_2_ nanoparticles showed that the nanoparticles were endocytosed by the cells. The concentration and time-dependent effects on viability of the SiO_2_ and TiO_2_ nanoparticles showed that both particles induced cytotoxicity at sub-3 mM concentrations. We have conducted an apoptosis assay using 1 mM of SiO_2_ and TiO_2_ nanoparticles as the viabilities of both the 2D and 3D CCD-18Co cultures decreased >30% at this concentration. The SiO_2_ and TiO_2_ nanoparticles also increased the % of 2D CCD-18Co cells undergoing apoptosis, compared to that of the untreated control cells. We also tested whether the SiO_2_ and TiO_2_ nanoparticles induced toxicity in 3D CCD-18Co spheroids. Similar to the results in the 2D cells, the SiO_2_ and TiO_2_ nanoparticles induced cytotoxicity in the 3D CCD-18Co spheroids in both a concentration- and time-dependent manner. Interestingly, the IC_50_ values of SiO_2_ nanoparticles in 2D and 3D CCD-18Co cells only differed by 37.5%, while the difference for the TiO_2_ nanoparticles was 227.3%. We speculate that the slightly larger TiO_2_ nanoparticles may affect the endocytosis process in the 3D CCD-18Co spheroids, resulting in reduced cytotoxicity. We next evaluated the SiO_2_ and TiO_2_ nanoparticle-mediated in vivo toxicities. ICR mice were orally fed 300 and 2000 mg/kg, and the data suggested that both oral doses do not significantly affect body weight or cause the deaths of the mice. Our in vivo acute oral toxicity results suggested that both the SiO_2_ and TiO_2_ nanoparticles are GHS grade 5 with LD_50_ values greater than 2000 mg/kg. According to reports of Van der Zande et al. [34], however, in vivo studies with rats found no acute toxicity due to nanoparticle ingestion, but fibrosis in the liver at high dosages. No change in the survival rate were observed in our in vivo acute toxicity study possibly due to various causes, such as immune responses, but there is still a possibility of potential damages to targeted cells or tissues. Therefore, we have recognized the need of evaluation for the potential toxicity of SiO_2_ and TiO_2_ nanoparticles by human colon organoids that can replace human organs.

We conducted SiO_2_ and TiO_2_ nanoparticle toxicity assessments with human colon organoids. The progressively developing organoid technology is reducing the gap between conventional 2D, 3D cells cultures and in vivo models. Especially, human organoid models can more accurately predict a variety of responses for humans, such as efficacy assessment, toxicity testing, and pharmacokinetic analysis. Although some limitations on human organoids, such as accurately modelling organ development and human diseases, have been proposed, human organoids are undoubtedly the best evaluation tool for predicting toxicity so far. In this study, similar to the results with the 2D and 3D CCD-18Co systems, incubation with SiO_2_ and TiO_2_ nanoparticles induced toxicity with structural damage, and a decrease in viability was observed in the human colon organoids. However, unlike the results for the 2D and 3D CCD-18Co cells, where no significant changes in the expression of apoptotic proteins were observed compared to those of the control, the SiO_2_ and TiO_2_ nanoparticles induced a significant increase in the Bax/Bcl-2 ratio in the colon organoids compared to the untreated organoids. Analysis of the apoptotic pathway-related genes of human colon organoids also showed an increase in Bax gene expression and a decrease in Bcl-2 gene expression after treatment with SiO_2_ and TiO_2_ nanoparticles. However, both nanoparticles not only upregulated key apoptotic genes, such as Bax, but also downregulated the expression of other major apoptotic genes, such as caspases 1, 6 and 9. The in vivo study of Fritsch-Decker et al. [35], upon oral exposure to nanoparticles, increased pro-inflammatory cytokine levels (IL-1β, IL-6, and TNF-α) detected in the colons. In addition, side effects by the interaction of nanoparticles with the various components of the human immune system (oral, mucosal, systemic or topical) after oral exposure were presented [36].

We are currently investigating the detailed mechanism of SiO_2_ and TiO_2_ nanoparticle-induced toxicity regarding changes in apoptosis-related gene expression. In summary, our data suggest that both SiO_2_ and TiO_2_ nanoparticles induced toxicity in 2D and 3D CCD-18Co cells as well as in human colon organoids with sub-2.5 mM IC_50_ values (except TiO_2_ nanoparticles in human colon organoids). As both SiO_2_ and TiO_2_ are widely used for food additives and supplements, special care should be taken when both compounds are used in nano-sized structures.

## 4. Materials and Methods

### 4.1. Experimental Section

#### Materials

SiO_2_ (10–20 nm particle size, 99.5% trace metals basis) and TiO_2_ (21 nm primary particle size, ≥99.5% trace metals basis) nanoparticles were purchased from Sigma-Aldrich (St. Louis, MO, USA). CCD18-Co (human normal colon fibroblast) was purchased from the American Tissue Type Collection (ATCC, Manassas, VA, USA). Human normal colon organoid and human colon organoid growth media were obtained from Organoid Sciences (Seongnam, Korea). Minimum essential medium (MEM), fetal bovine serum (FBS), Dulbecco’s phosphate-buffered saline (DPBS) and penicillin/streptomycin were purchased from Thermo Fisher Scientific (Waltham, MA, USA). CrlOri:CD1 (ICR) mice (male, 6~7 weeks) were obtained from Orient Bio, Inc. (Seongnam, Korea). WST-8 [2-(2-methoxy-4-nitrophenyl)-3-(4-nitrophenyl)-5-(2, 4-disulfophenyl)-2H-tetrazolium, monosodium salt] solution (Cell Counting Kit-8, CCK-8) was purchased from Dojindo Molecular Technologies, Inc. (Kumamoto, Japan). The CellTiter-Glo 3D cell viability assay kit was obtained from Promega (Madison, WI, USA). The RT^2^ profiler PCR array was purchased from Qiagen (Hilden, Germany).

### 4.2. SiO_2_ and TiO_2_ Nanoparticle Preparation and Characterization

SiO_2_ and TiO_2_ nanoparticle powders were autoclaved and UV-sterilized overnight. The sterilized SiO_2_ and TiO_2_ nanoparticles were suspended and ultra-sonicated (Bransonic ultrasonic cleaner 3210, 120 W, Branson Ultrasonic Corporation, Danbury, CT, USA) for 15 min in pure ethanol (99.9%, Samchun Chemicals, Seoul, Korea). The nanoparticle suspensions underwent overnight UV sterilization. Scanning electron microscopy (SEM) images of the SiO_2_ and TiO_2_ nanoparticles were obtained by JSM-6700F (JEOL, Ltd., Tokyo, Japan). Transmission electron microscopy images of the nanoparticles and the cells with nanoparticles were obtained with Tecnai G2-T20S and Themis TEM (Thermo Fisher Scientific, Inc.). Photoluminescence spectra of 1 µM SiO_2_ and TiO_2_ nanoparticle suspensions were obtained with an LS-55 fluorescence spectrometer (PerkinElmer, Inc., Waltham, MA, USA). The UV-visible absorbance spectra and Fourier transform infrared spectra (FT-IR) of 1 mM SiO_2_ and TiO_2_ nanoparticle suspensions were obtained with a UV-2550 spectrophotometer (Shimadzu, Kyoto, Japan) and Nicolet 5700 (Thermo Fisher Scientific, Inc.). The hydrodynamic diameters, polydispersity indexes and zeta potentials of SiO_2_ and TiO_2_ nanoparticles were analyzed with Litesizer 500 (Anton Paar, Graz, Austria).

### 4.3. Incubation of 2D and 3D CCD-18Co and Human Colon Organoids with SiO_2_ and TiO_2_ Nanoparticles and Assessment of Cell Viability

CCD-18Co cells (2000) were seeded on 96-well cell culture plates (Corning, Corning, NY, USA) with 100 µL of MEM supplemented with 10% FBS and 1% penicillin-streptomycin. After overnight incubation, designated concentrations (0.001, 0.01, 0.05, 0.2, 0.5, 1, 2, 5, 20 mM) of SiO_2_ and TiO_2_ nanoparticles were co-incubated with the cells for designated periods (6, 12, 24 and 48 h). The cells were washed with DPBS twice, and 100 µL of fresh MEM containing 10% CCK-8 was added and incubated for 1 h. Absorbance at 450 nm was monitored and recorded (SpectraMax M5e microplate reader, Molecular Devices, San Jose, CA, USA). The rate of cells undergoing apoptosis was monitored with an apoptosis detection kit (Millipore Sigma, Burlington, MA, USA). For the 3D CCD-18Co spheroids, 2000 cells in 100 µL of media were seeded on 96-well clear round bottom ultralow attachment microplates (Corning) and centrifuged at 200 g for 5 min. After SiO_2_ and TiO_2_ nanoparticle treatment, the CellTiter-Glo 3D cell viability assay was performed and the luminescent signal was monitored. For human colon organoid culture, human normal colon organoids were suspended in an ice-cold mixture of 40% human colon organoid growth media and 60% matrigel (Corning). The mixture containing the crypts was plated in a 96-well plate that had been previously incubated at 37 °C to create dome structures and immediately incubated at 37 °C for 10 min. The wells were then filled with human colon organoid growth media and cultured at 37 °C with 3 medium changes per week. Human organoids were passaged every 7–10 days by dissociation in Gentle Cell Dissociation Reagent for 15 min and plated in 10 µL matrigel domes at an approximate density of 200 organoids per well. Human colon organoids on 96-well cell culture plates were treated with the designated concentrations of SiO_2_ and TiO_2_ nanoparticles. After 48 h of treatment, the human colon organoids were washed and further treated with CellTiter-Glo 3D cell viability assay reagent following the manufacturer’s protocol. The luminescence intensity from the human colon organoids treated with the SiO_2_ and TiO_2_ nanoparticles was monitored with a UV-2550 spectrophotometer.

### 4.4. The 2D/3D CCD-18Co and Human Colon Organoid Imaging

Brightfield images of the 3D CCD-18Co spheroids were obtained using an Eclipse Ti2 inverted microscope (Nikon, Tokyo, Japan). For live–dead imaging, 2D and 3D CCD-18Co cells with the control SiO_2_ and TiO_2_ treatments were incubated with 2 µM calcein-AM cell permeant dye (for live cell staining, Thermo Fisher Scientific, Inc.) and 4 µM ethidium homodimer-1 (EthD-1, for dead cell staining; Thermo Fisher Scientific, Inc.) for 5 min, and washed with DPBS 5 times. Fluorescence images were obtained with an Eclipse Ti2 microscope. For human colon organoid bright field imaging after SiO_2_ and TiO_2_ treatment, a Lionheart FX (BioTek Instruments, Inc., Winooski, VT, USA) automated imaging protocol was used. For live fluorescence imaging, human colon organoids after SiO_2_ and TiO_2_ treatment were incubated with 2 µM calcein-AM and 4 µM EthD-1.

### 4.5. PCR Arrays

Total RNA isolation from human colon organoids preserved in RNAlater was performed using the RNeasy mini prep Kit (Qiagen) according to the manufacturer’s protocols. The quantity and quality of the RNA samples were determined using a NanoDrop 2000 (Thermo Fisher Scientific, Inc.). A total of 1000 ng of RNA was reverse transcribed to cDNA using the RT2 First Strand Kit (Qiagen). The RT^2^ Profiler™ PCR Array Human Cell Death Pathway Finder (PAHS-212Z, Qiagen), using SYBR Green chemistry (Qiagen), was used to evaluate the expression of 84 key genes, according to the manufacturer’s protocol, on the Rotor-Gene Q (Qiagen). The expression levels of each gene were normalized to the geometric mean values of housekeeping genes (GAPDH), based on the RefFinder algorithm (www.qiagen.com/shop/genes-and-pathways/dataanalysis-center-overview-page).

### 4.6. Western Blot

After 24 h incubation with 1 mM SiO_2_ and TiO_2_ nanoparticles, 2D and 3D CCD-18Co cells were lysed with 4 °C whole-cell extract buffer (pH 7.4) with protease inhibitors. The concentrations of the proteins were determined with a BCA protein assay kit (Thermo Fisher Scientific, Inc.). Twenty micrograms of lysed protein was subjected to electrophoresis using 10% sodium dodecyl sulfate-polyacrylamide gels (Bio-Rad Laboratories, Inc., Hercules, CA, USA). After the transfer to polyvinylidene fluoride membranes (PVDF membranes, Bio-Rad Laboratories, Inc.), the PVDF membranes were blocked with 5% bovine serum albumin (Sigma-Aldrich) for 1 h at room temperature. Western blotting was performed by incubation with primary antibody (1:1000 dilution) in 1x casein blocking buffer (Sigma-Aldrich) overnight at 4 °C, followed by horseradish peroxidase (HRP)-conjugated secondary antibody (1:1000 in Tris-buffered saline, 0.1% Tween^®^ 20 detergent (TBST), Thermo Fisher Scientific, Inc.) addition and further incubation at room temperature for 2 h. The protein bands were visualized by a Chemidoc XRS+ imaging system (Bio-Rad Laboratories, Inc.). ß-actin (Cell Signaling Technology, Inc. (CST), Danvers, MA, USA) was used as the protein loading control. The following primary antibodies were used in the experiments: BAX (CST), Bcl-2 (CST), and cytochrome C (CST).

### 4.7. In Vivo ICR Mice Toxicity Study

All animal experiments were carried out using CD-1(ICR) mice according to the established guidelines of the Institutional Animal Care and Use committee of the Korea Research Institute of Chemical Technology. All animals were maintained under a room illuminated daily from 07:00 to 19:00 (12:12 h light/dark cycle), with a temperature of 23 ± 1 °C, a ventilation of 10–12 times per hour, and a humidity of 55 ± 5%. Mice were caged individually and allowed free access to tap water and feed. For SiO_2_ and TiO_2_ nanoparticle acute oral toxicity assessment, in vivo experiments with ICR mice were conducted based on the OECD Guidelines for the Testing of Chemicals Section 4 Health Effects Test No. 423 Acute Oral Toxicity-Acute Toxic Class Method (17 December 2001). In brief, ICR mice were stabilized for 7 days and fasted for 16 h immediately before oral feeding with 300 and 2000 mg/kg SiO_2_ and TiO_2_ nanoparticles in 0.5% CMC solution (Sigma-Aldrich). Body weight and % survival rate of the SiO_2_ and TiO_2_ nanoparticle-fed mice were monitored. All in vivo experimental procedures were approved by the Animal Research Committee of the Korea Research Institute of Chemical Technology (approval number: 2020-7A-05-06).

### 4.8. Statistical Analysis

Statistical analysis was performed using GraphPad Prism (version 6; GraphPad Software, Inc., San Diego, CA, USA). Each experiment was performed in triplicate, and values are expressed as the mean ± standard deviation. Statistical significance was examined by a *t*-test or one-way or 2-way analysis of variance. Statistical significance is denoted as * for *p* < 0.05 and ** for *p* < 0.01.

## Supplementary Materials

The following are available online. Table S1: Hydrodynamic diameter and polydispersity index of SiO_2_ and TiO_2_ nanoparticles; Table S2: Zeta potential of SiO_2_ and TiO_2_ nanoparticles.
